# Neurotoxicity of Unconjugated Bilirubin in Neonatal Hypoxic-Ischemic Brain Injury *in vitro*

**DOI:** 10.3389/fped.2021.659477

**Published:** 2021-04-20

**Authors:** Carlo Dani, Simone Pratesi, Guido Mannaioni, Elisabetta Gerace

**Affiliations:** ^1^Division of Neonatology, Careggi University Hospital of Florence, Florence, Italy; ^2^Department of Neurosciences, Psychology, Drug Research and Child Health, University of Florence, Florence, Italy

**Keywords:** bilirubin, neurotoxicity, hypoxia-ischemia, ROS, pioglitazone, allopurinol, organotypic slice

## Abstract

**Background:** The pathophysiology of bilirubin neurotoxicity in course of hypoxic–ischemic encephalopathy (HIE) in term and preterm infants is still poorly understood. We hypothesized that oxidative stress may be a common mechanism that link hyperbilirubinemia and HIE.

**Objectives:** The objective of the present study was to evaluate whether unconjugated bilirubin (UCB) may enhance the HI brain injury by increasing oxidative stress and to test pioglitazone and allopurinol as new antioxidant therapeutic drugs *in vitro*.

**Methods:** The effects of UCB were tested on organotypic hippocampal slices subjected to 30 min oxygen-glucose deprivation (OGD), used as *in vitro* model of HIE. The experiments were performed on mature (14 days in culture) and immature (7 days in culture) slices, to mimic the brains of term and preterm infants, respectively. Mature and immature slices were exposed to UCB, human serum albumin (HSA), pioglitazone, and/or allopurinol for 24 h, immediately after 30 min OGD. Neuronal injury was assessed using propidium iodide (PI) fluorescence. ROS formation was quantified by using the 2′,7′-dichlorodihydrofluorescein diacetate (DCF-DA) method.

**Results:** In mature slices, we found that the neurotoxicity, as well as oxidative stress, induced by OGD were enhanced by UCB. HSA significantly prevented UCB-increased neurotoxicity, but had a slight reduction on ROS production. Allopurinol, but not pioglitazone, significantly reduced UCB-increased neurotoxicity induced by OGD. In immature slices exposed to OGD, no increase of neuronal death was observed, whereas oxidative stress was detected after UCB exposure. HSA, pioglitazone and allopurinol have no protective effects on both OGD-induced neuronal death and on UCB-induced oxidative stress. For this reason, UCB, pioglitazone and allopurinol was also tested on ischemic preconditioning protocol. We found that UCB abolished the neuroprotection induced by preconditioning and increased oxidative stress. These effects were restored by allopurinol but not pioglitazone.

**Conclusions:** UCB characterized a different path of neuronal damage and oxidative stress in mature and immature hippocampal slice model of HIE. Management of hyperbilirubinemia in a complex pathological condition, such as HIE and hyperbilirubinemia, should be very careful. Allopurinol could deserve attention as a novel pharmacological intervention for hyperbilirubinemia and HIE.

## Introduction

Hypoxic–ischemic encephalopathy (HIE) is a clinical syndrome that affects newborns following severe or prolonged cerebral hypoxic–ischemic episodes before or during birth. It was reported that in developed countries its incidence ranges from one to eight per 1,000 live births and that is higher in undeveloped countries (about 26 per 1,000 live births) (1). Infants affected by HIE have an augmented risk of death and lifelong disability, such as cerebral palsy, cognitive impairment, developmental delay, visual and learning disability, and epilepsy (2, 3). Nowadays, the standard therapy for infants diagnosed with moderate-to-severe HIE is hypothermia (4), although its efficacy is only partial and almost 50% of treated infants present adverse outcomes (5).

The pathophysiology of HIE is complicated and involves acute effects, such as cell energy failure caused by hypoxia and low level of glucose, and delayed effects, such as extracellular accumulation of excitatory neurotransmitters, that activating apoptotic or necrotic cascades lead to cell death (6, 7). Many studies have been described the role of oxidative stress in the pathophysiology of HIE and reactive oxygen species (ROS) are believed to contribute to the degenerative processes of the tissues that lead to acute and chronic brain injury (8, 9).

It has been suggested that bilirubin is involved in the balance between pro-oxidant and antioxidant agents of newborns, which is particularly challenged in the transitional postnatal period. Despite the role of bilirubin as antioxidant, as well as scavenger of ROS, is well-documented in *in vitro* [(10–13)] and *in vivo* animal studies (14), its role in newborns with or without jaundice is still debated (15–20). The potential neuroprotective role of bilirubin, as antioxidant agent, in infants with HIE remains poorly investigated. We have recently showed that infants with moderate-to-severe HIE present lower values of peak and mean total serum bilirubin (TSB) as compared to control infants, and speculated that this might be due to hypoxic repression of heme-oxygenase (HO) (21). This results seems to exclude that increased levels of TSB may act as neuroprotective antioxidant agent in infants with HIE and, conversely, might happen to protect infants who have suffered HIE from further brain injury from hyperbilirubinemia (21).

Intermittent hypoxic (IH) events are common in extremely preterm infants and it was suggested a relationship between IH and ROS (22). Moreover, IH could contribute to neuroprotection against profound hypoxemic events (23). This is what occur after preconditioning, an endogenous neuroprotective mechanism by which exposure to a mild preconditioning stress (such as IH) results in resistance to a consequent lethal ischemic insult. The neuroprotection induced by preconditioning acts with several mechanisms, which include cellular and molecular events that counteracts cell death, including oxidative stress (24).

Many studies have been shown that two drugs, the PPAR-γ agonist pioglitazone and the xanthine oxidase inhibitor allopurinol, can attenuate neurodegenerative and neuroinflammatory processes in the brain. In particular, it was recently demonstrated in animal experiments that these drugs confer neuroprotection against cerebral ischemia, by activating several mechanisms that involve both anti-oxidant and anti-inflammatory effects (25, 26). Most importantly, the therapeutic potential of pioglitazone has been shown in patients after ischemic stroke or transient ischemic attack events (27) and allopurinol has been recently tested in neonates for HIE (28). In addition, the beneficial action of pioglitazone was also evidentiated in models of spinal cord injury (29) and amyotrophic lateral sclerosis (30), demonstrating their neuroprotective effect. Futhermore, allopurinol has been recently proposed as a new pharmacological intervention for neonatal brain injury and in particular for neonatal asphyxia (31–33).

Thus, some potential determinants of bilirubin neurotoxicity in course of cerebral hypoxic–ischemic episodes are particularly interesting from a clinical point of view, for example the maturity or immaturity of exposed tissues, the binding to its endogenous ligand HSA, the possible interaction with antioxidant and/or anti-inflammatory drugs, such as pioglitazone (25) and allopurinol (26), and the effects of UCB on neuroprotection induced by preconditioning (23). Therefore, we hypothesized that UCB might exacerbate the hypoxic/ischemic (HI) brain injury by increasing oxidative stress and that HSA, pioglitazone, allopurinol, and preconditioning might limit this effect through their antioxidant properties. To assess this hypothesis, we carried out this *in vitro* study in rat organotypic hippocampal slices which were subjected to oxygen-glucose deprivation (OGD) to mimic neonatal HIE.

## Materials and Methods

Male and female Wistar rats were obtained from Charles River (MI, Italy). Animals were housed at 23 ± 1°C under a 12 h light–dark cycle (lights on at 07:00) and were fed a standard laboratory diet with *ad libitum* access to water. The experimental protocols were approved by the Italian Ministry of Health (Aut. 176; 17E9C.N.VAS) and the European Communities Council Directive of 2010/63/EU. The authors further attest that all efforts were made to minimize the number of animals used and their suffering.

### Materials

Unconjiugated Bilirubin (UCB), human serum albumin (HSA), pioglitazone hydrochloride, allopurinol, propidium iodide (PI) and 2,7-dichlorofluorescein diacetate (DCFH2-DA) were purchased from Sigma (St Louis, MO, USA). Tissue culture reagents were obtained from Gibco-BRL (San Giuliano Milanese, MI, Italy) and Sigma (St Louis, MO, USA).

## Methods

### Preparation of Rat Organotypic Hippocampal Slice Cultures

Organotypic hippocampal slice cultures were obtained from the brains of male and female Wistar rat pups (Harlan, Milan, Italy) as previously reported (34, 35). Briefly, hippocampi were isolated and removed from the brains of Wistar rat pups. Transverse slices (420 μm) were prepared using a McIlwain tissue chopper and transferred onto semi-porous membranes inserts (Millicell-CM, catalog number: #PICM03050; Millipore, Italy), in six well tissue culture plates containing 1.2 ml medium per well. Slices were maintained at 37°C in an incubator in an atmosphere of humidified air and 5% CO_2_ for 14 days (mature slices) or 7 days (immature slices) before experiments, in order to mimic what may occur in term and preterm infants, as previously reported in Gerace et al. (36) and Dani et al. (37, 38). The time of maturation *in vitro* was established according to recent studies, asserting that the first postnatal week in the rat corresponds to 19–28 weeks of gestation in humans (39, 40). Moreover, a previous paper of our laboratory have demonstrated that hippocampal slices exhibit increasing protein and receptor expression, as well as increasing electrical activity, during maturation *in vitro* mimicking what occur *in vivo* animals (36). All experiments were performed in primary slice cultures obtained from different litters. Before experiments all the slices were screened for viability by phase-contrast microscopy analysis; slices displaying signs of neurodegeneration were discarded from the study.

### UCB Exposure and Drug Treatment in Organotypic Hippocampal Slices

Organotypic hippocampal slice were exposed for 24 h to UCB (100 μM, corresponding to 5.850 mg/dl), or UCB plus pioglitazone (10 μM) or allopurinol (10 μM) after 7 days (immature) or 14 days (mature) of culture *in vitro* as previously reported (37, 38). After drug treatments, the slices were assessed for neuronal injury using PI fluorescence. All the experiments with UCB were performed under light protection to avoid photodegradation. The calculated unbound fraction of UCB was 215 nM at 100 equimolar concentrations of UCB and HSA, according to the model [K^′^_*f*_= B_t_–B_f_/B_f_ (HSA–B_t_+B_f_); K^′^_*f*_ is the binding constant of albumin; B_t_ is the total bilirubin concentration; B_f_ is the free bilirubin concentration)] proposed by Weisiger et al. (41) and Ostrow et al. (42). Thus, our model generally reproduced concentrations of unbound bilirubin which are associated to the development of kernicterus in preterm (15–34 nM) and in term (>68 nM) infants (43).

### OGD Protocol in Rat Organotypic Hippocampal Slices

Cultures were exposed to oxygen and glucose deprivation (OGD) in order to mimic what occur after HI episodes and HIE, as previously reported in detail (35, 44–46). Briefly, OGD was reproduced by exposing the slices to serum- and glucose-free medium saturated with 95% N_2_ and 5% CO_2_ for 30 min at 37°C in an airtight anoxic chamber equipped with an oxygen gas controller (BioSpherix, New York, USA). The cultures were then transferred to oxygenated serum-free medium (75% Eagle's minimal essential medium; 25% Hank's balanced salt solution; 2 mM l-glutamine; and 3.75 μg/ml amphotericin B) containing 5 mg/ml glucose and returned to the incubator under normoxic conditions. UCB (100 μM, corresponding to 5.850 mg/dl), HSA (100 μM, corresponding to 0.664 g/dL), pioglitazone (10 μM) and allopurinol (10 μM) were added to the culture medium immediately after 30 min OGD and were kept in culture until neuronal injury was evaluated 24 h later.

### Ischemic Preconditioning Protocol in Organotypic Hippocampal Slices

Ischemic preconditioning was obtained by exposing the slices to 10 min OGD, as previously described in detail (35, 45, 46). Immature hippocampal slices were exposed to ischemic preconditioning (10 min OGD) or to ischemic preconditioning plus UCB (100 μM) alone or plus pioglitazone (10 μM) or allopurinol (10 μM) for the subsequent 24 h, before exposure to 30 min OGD toxic insult. Neuronal injury was evaluated 24 h later by measuring the intensity of PI fluorescence.

### Assessment of CA1 Pyramidal Cell Injury

In all experiments, neuronal damage was assessed in CA1 pyramidal cells using the PI fluorescence method as reported in Gerace et al. (35), Gerace et al., (45, 46), and Dani et al. (37, 38). PI (5 μg/ml) was added to the medium either at the end of the experiments. Thirty minutes later, fluorescence was viewed using an inverted fluorescence microscope (Olympus IX-50; Solent Scientific, Segensworth, UK) equipped with a xenon-arc lamp, a low-power objective (4X) and a rhodamine filter. Images were digitized using a video image obtained by a CCD camera (Diagnostic Instruments Inc., Sterling Heights, MI, USA) controlled by software (InCyt Im1^TM^; Intracellular Imaging Inc., Cincinnati, OH, USA) and subsequently analyzed using the Image-Pro Plus morphometric analysis software (Media Cybernetics, Silver Spring, MD, USA). In order to quantify cell death, the CA1 hippocampal subfield was identified and encompassed in a frame using the drawing function in the image software (ImageJ; NIH, Bethesda, USA) and the optical density of PI fluorescence was detected. There was a linear correlation between CA1 PI fluorescence and the number of injured CA1 pyramidal cells as detected by morphological criteria (47).

### Reactive Oxygen Species Production

In all experiments, oxidative stress and ROS production was assessed in organotypic hippocampal slices using the 2′,7′-dichlorodihydrofluorescein diacetate (DCFH2-DA) fluorescent probe, as reported in Gerace et al. (45, 46). Briefly, at the end of the experiments, hippocampal slices were incubated with 10 mM DCFH2-DA at 37C for 30 min. Fluorescence was viewed 30 min later using an inverted fluorescence microscope (Olympus IX-50; Solent Scientific, Segensworth, UK) equipped with a xenon-arc lamp and a rhodamine filter (excitation 450 and 490 nm, emission 520 nm). Images were digitized using a video image obtained by a CCD camera (Diagnostic Instruments Inc., Sterling Heights, MI, USA) controlled by software (InCyt Im1; Intracellular Imaging Inc., Cincinnati, OH, USA) and subsequently analyzed using the Image-Pro Plus morphometric analysis software (Media Cybernetics, Silver Spring, MD, USA). In order to quantify ROS production, hippocampal subfield was encompassed in a frame using the drawing function in the image software (ImageJ; NIH, Bethesda, USA) and the optical density of DCFH2-DA fluorescence was detected (45, 46).

### Statistical Analysis

Data are presented as means ± SEM of n experiments. Quantitative analysis are expressed as percentage of control (CRL) PI or DCF fluorescence. Each experimental point consisted of 28–48 hippocampal slices. Statistical significance of differences between PI or DCFH_2_ fluorescence intensities was evaluated by performing one-way analysis of variance (ANOVA) followed by Tukey's *w* test for multiple comparisons. All statistical calculations were performed using GraphPad Prism v.5 for Windows (GraphPad Software, San Diego, CA). A *P* < 0.05 was considered statistically significant.

## Results

### Effects of HSA on UCB-Induced-Toxicity and ROS Production in OGD Mature Organotypic Hippocampal Slices

**Figure 1:**
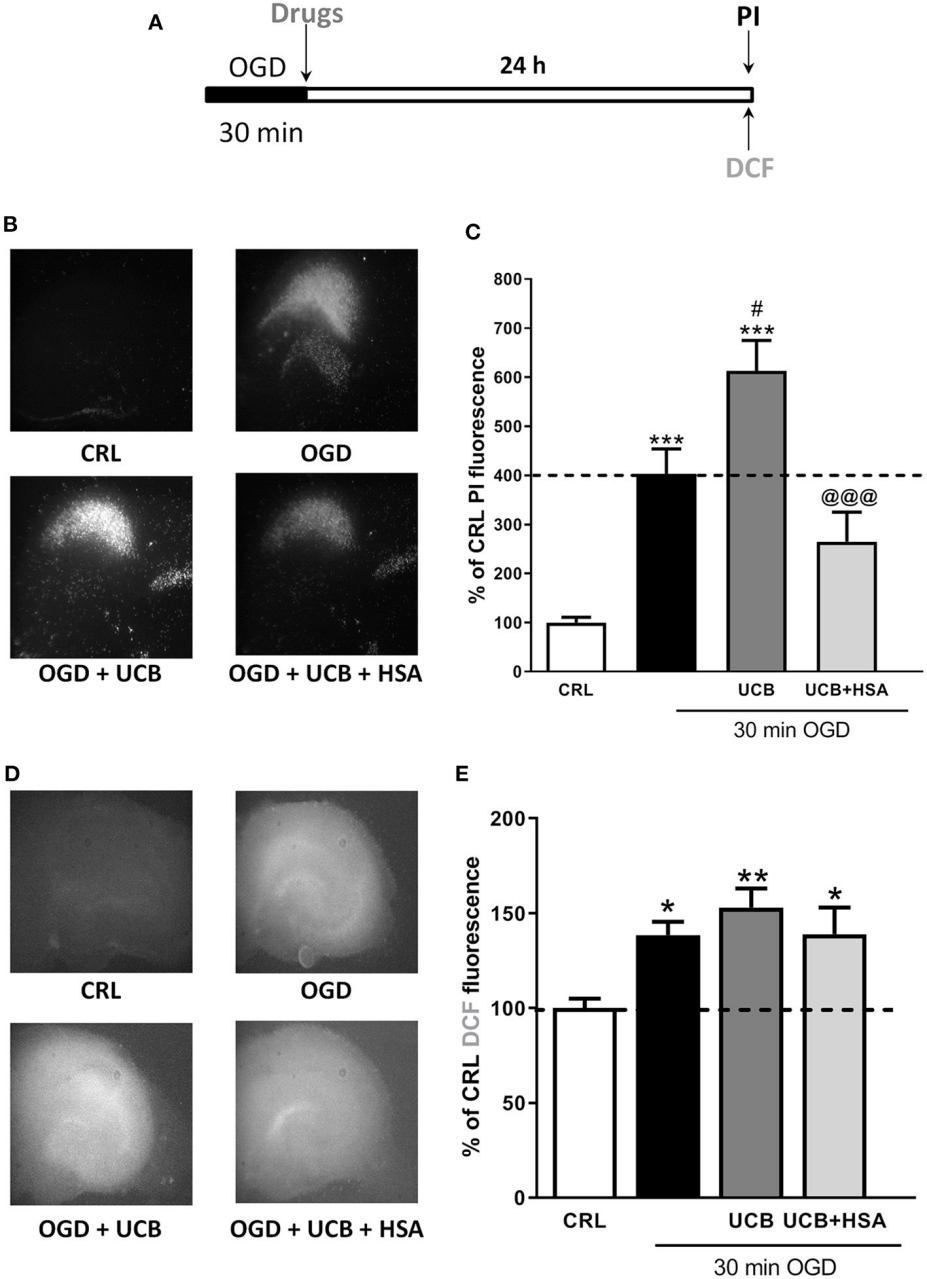
Effects of HSA on UCB-induced-toxicity and ROS production in OGD mature organotypic hippocampal slices. **(A)** Experimental protocol showing mature hippocampal slices exposed to 100 μM of UCB alone or plus HSA (100 μM) for 24 h immediately after 30 min OGD. **(B)** Qualitative analysis shows that the CA1 damage induced by OGD is enhanced by UCB whose effect is abolished by equimolar concentration of HSA. **(C)** Quantitative analysis of PI fluorescence: Bars represent the mean ± SEM of at least seven experiments (about ≥28 slices for each experimental point). *P < 0.05 vs. CRL; **P < 0.01 vs. CRL; ***P < 0.001 vs. CRL; ^#^*P* < 0.05 vs. OGD; ^@@@^*P* < 0.001 vs. UCB. **(D)** Qualitative analysis shows that oxidative stress induced by OGD is enhanced by UCB (100 μM), whose effect is abolished by equimolar concentration of HSA. **(E)** Quantitative analysis of DCFH2-DA fluorescence: Bars represent the mean ± SEM of at least seven experiments (about ≥28 slices for each experimental point). *P < 0.05 vs. CRL; **P < 0.01 vs. CRL.

Mature organotypic hippocampal slices were exposed to UCB (100 μM) for 24 h immediately after 30 min OGD (Figure 1A), to resemble what could occur in term infants with HIE who develop jaundice. In this condition, OGD induces CA1 pyramidal cell injury (Figure 1C black column) and cellular oxidative stress (Figure 1E black column). The neurotoxicity induced by OGD was enhanced by UCB; this effect was reduced by the simultaneous addition of an equimolar amount of HSA (100 μM) (Figure 1B,C). Conversely, the pro-oxidant effect provoked by OGD was mildly enhanced by UCB and the addiction of 100 μM HSA slightly reduced UCB-increased ROS production (Figure 1D,E).

### Effects of Pioglitazone and Allopurinol on UCB-Induced-Toxicity and ROS Production in OGD Mature Organotypic Hippocampal Slices

**Figure 2:**
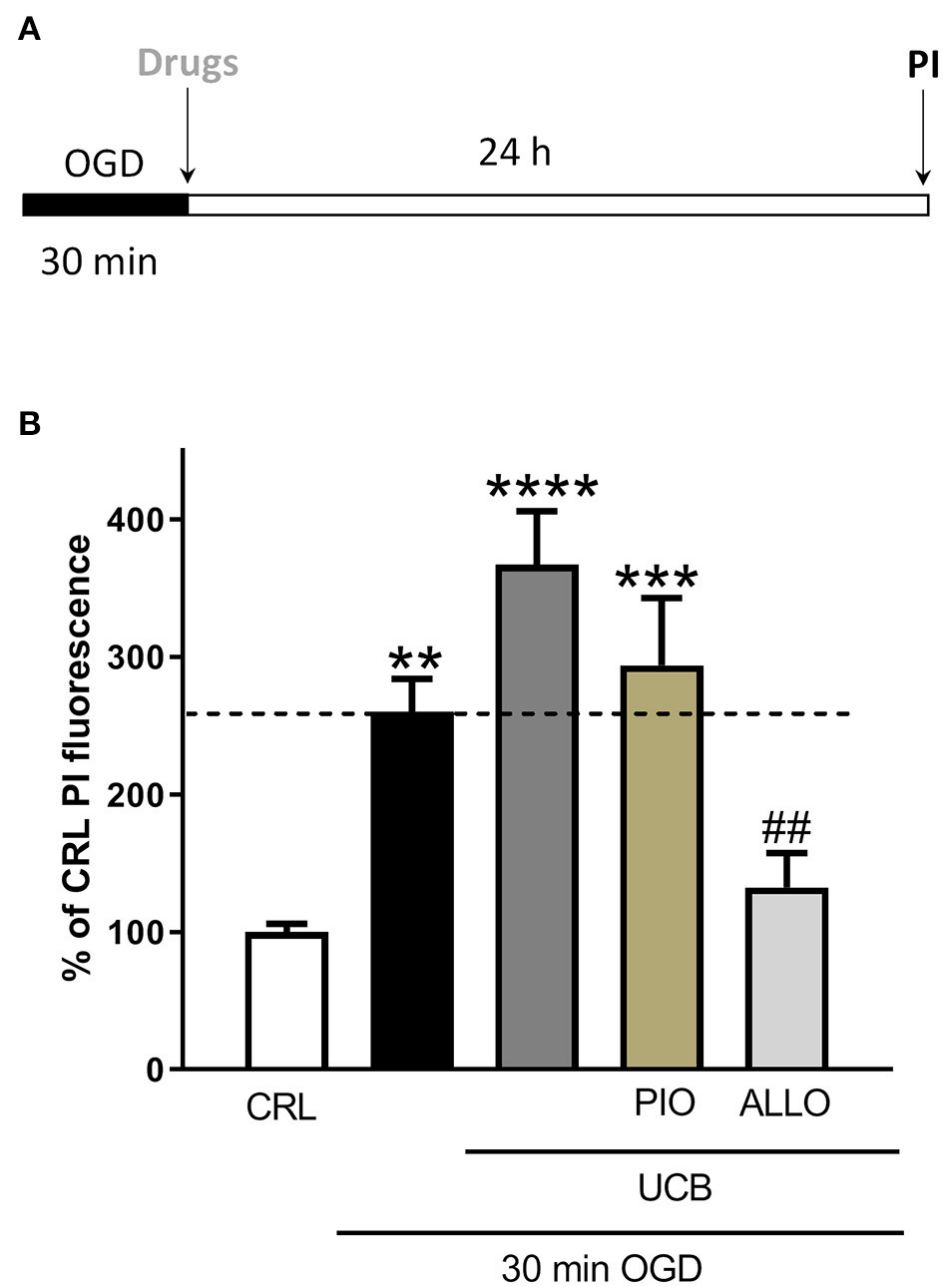
Effects of pioglitazone and allopurinol on unconjugated bilirubin (UCB)-induced-toxicity in OGD mature organotypic hippocampal slices. **(A)** Experimental protocol showing mature hippocampal slices subjected to 30 min OGD and then to UCB (100) and pioglitazone (10 μM) or allopurinol (10 μM) for 24 h. **(B)** Quantitative analysis shows that the addition of allopurinol but not pioglitazone reduces cellular death induced by UCB, measured by the intensity of PI fluorescence. Bars represent the mean ± SEM of at least seven experiments (about ≥44 slices for each experimental point). **P < 0.01 vs. CRL; ***P < 0.001 vs. CRL; ****P < 0.0001 vs. CRL; ^##^*P* < 0.01 vs. UCB.

In order to evaluate whether antioxidant pioglitazone and allopurinol could be able to attenuate the neuronal injury and oxidative stress induced by UCB in OGD mature slices, we firstly tested separately both pioglitazone and allopurinol in OGD- or UCB-induced neurotoxicity. For these experiments, pioglitazone or allopurinol were added to the incubation medium immediately after 30 min OGD and maintained for the following 24 h or co-incubated with UCB for 24 h. We observed that the addition of 10 μM pioglitazone decreased cellular death in OGD but not in UCB exposed slices and it was ineffective in decreasing cellular oxidative stress in both experimental setting, suggesting that its neuroprotection was not mediated by ROS production (Supplementary Figure 1). Conversely, the addition of 10 μM allopurinol decreased OGD and UCB-induced cellular death, as well as oxidative stress, in UCB exposed slices (Supplementary Figures 2A,C,D), while no significant reduction was noted for oxidative stress after OGD (Supplementary Figure 2B). Starting from these data, we studied the effects of pioglitazone and allopurinol on UCB-induced-toxicity in mature hippocampal slices exposed to OGD. For these experiments, the drugs were added to the incubation medium together with UCB immediately after 30 min OGD and maintained for the subsequent 24 h. Our results show that allopurinol but not pioglitazone was able to significantly reduce UCB-increased neurotoxicity in mature slices exposed to OGD, ascribing its efficacy through ROS production reduction (Figure 2).

### Effects of HSA on UCB-Induced-Toxicity and ROS Production in OGD Immature Organotypic Hippocampal Slices

**Figure 3:**
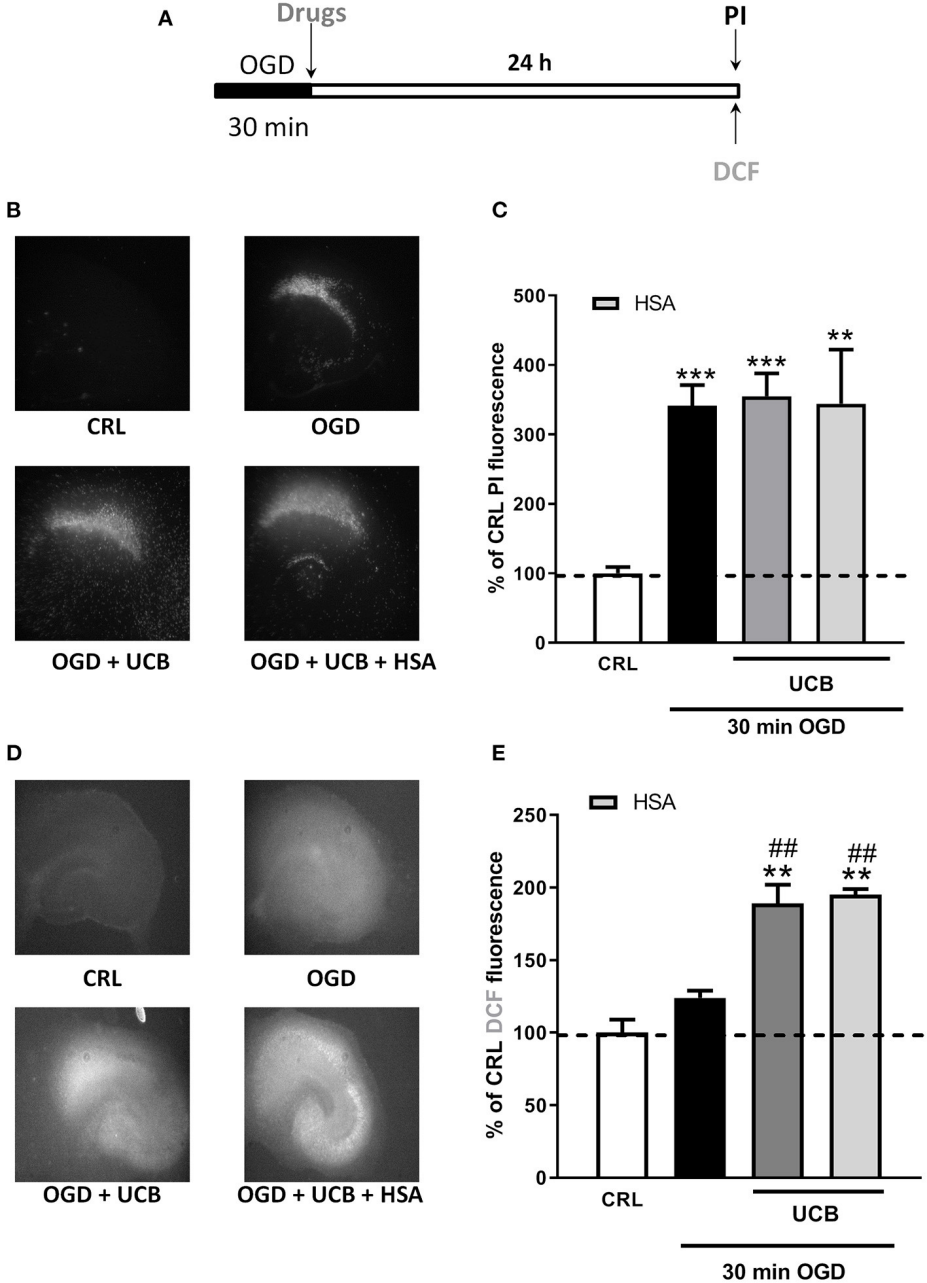
Effects of HSA on UCB-induced-toxicity and ROS production in OGD immature organotypic hippocampal slices. **(A)** Experimental protocol showing immature hippocampal slices exposed to UCB (100 μM) alone or plus HSA (100 μM) for 24 h, immediately after 30 min OGD. **(B)** Qualitative analysis shows that CA1 damage induced by OGD is unchanged by UCB and HSA. **(C)** Quantitative analysis of PI fluorescence: Bars represent the mean ± SEM of at least seven experiments (about ≥38 slices for each experimental point). **P < 0.01 vs. CRL; ***P < 0.001 vs. CRL. **(D)** Qualitative analysis shows that OGD did not induce oxidative stress, while the addition of UCB (100 μM) increases ROS production that is unchanged by HSA. **(E)** Quantitative analysis of DCFH2-DA fluorescence: Bars represent the mean ± SEM of at least seven experiments (about ≥24 slices for each experimental point). **P < 0.01 vs. CRL; ^##^*P* < 0.01 vs. OGD.

**Figure 4:**
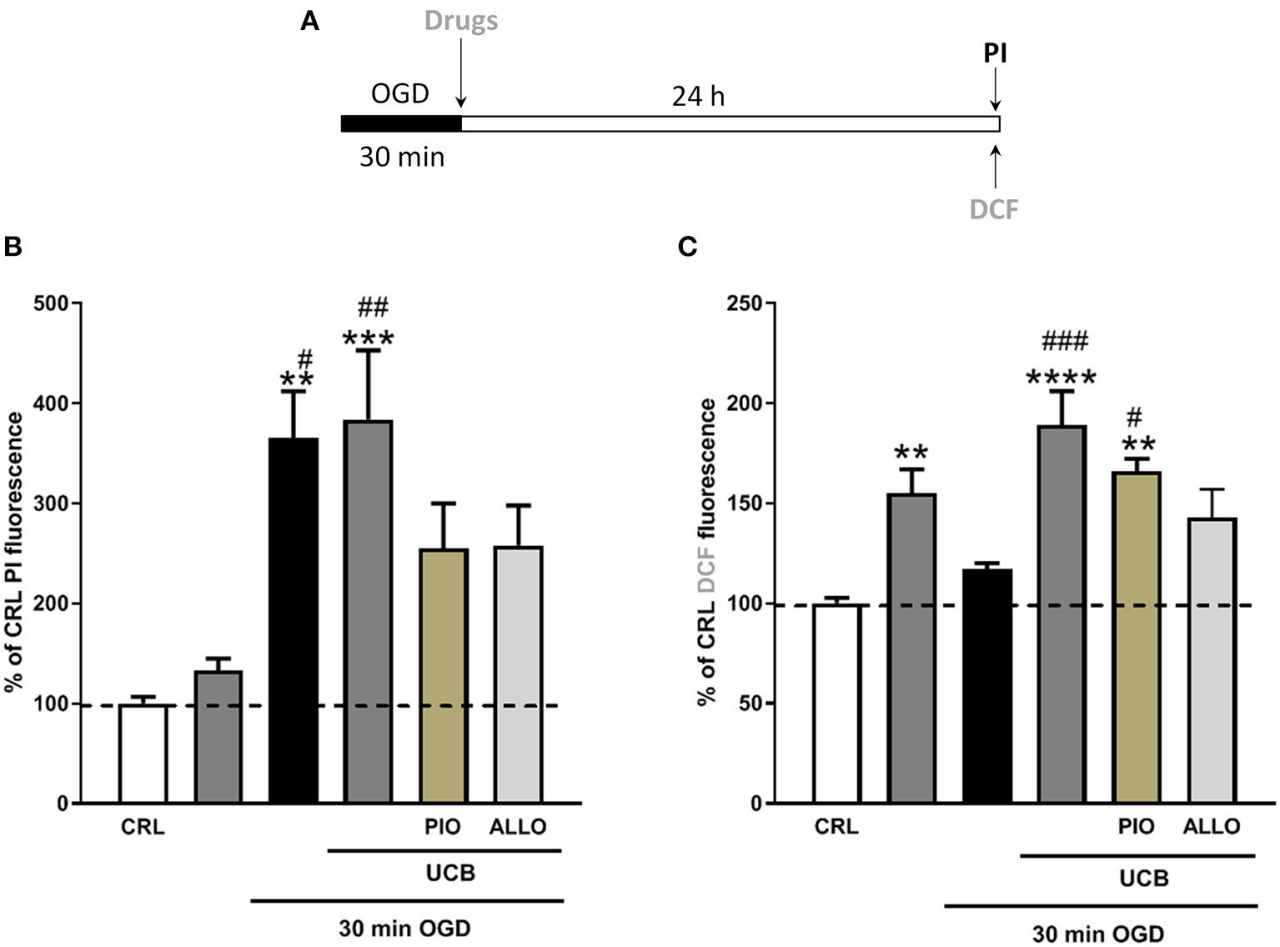
Effects of pioglitazone and allopurinol on unconjugated bilirubin (UCB) induced-toxicity in OGD immature organotypic hippocampal slices. **(A)** Experimental protocol showing immaure hippocampal slices exposed to UCB (100 μM) alone or plus pioglitazone and allopurinol (10 μM) for 24 h, immediately after 30 min OGD. **(B)** Quantitative analysis shows that pioglitazone and allopurinol have a trend to decrease cellular death in UCB exposed OGD slices. **(C)** Differently, OGD did not increase cellular oxidative stress that was increased by UCB and decreased by allopurinol but not pioglitazone. Quantitative analysis of PI and DCFH2-DA fluorescence: Bars represent the mean ± SEM of at least seven experiments (about ≥48 slices for each experimental point). **P < 0.05 vs. CRL; ***P < 0.01 vs. CRL; ^#^*P* < 0.05 vs. UCB; ^##^*P* < 0.01 vs. OGD; **** < 0.001 vs CRL; ^###^ < 0.01 vs OGD.

We used immature organotypic hippocampal slices exposed to 100 μM UCB for 24 h immediately after OGD, to resemble what could occur in preterm infants with HIE who develop jaundice. As well as for mature slices, exposure of immature slices to 30 min OGD lead to selective damage of CA1 pyramidal cells, but in this case was not affected by the addition of UCB nor by the further addition of equimolar amount of HSA (Figure 3B). Differently, exposure of immature slices to OGD did not promote cellular oxidative stress, which was induced by the next addition of UCB (Figure 3E). Interestingly, the addiction of UCB alone promote ROS formation but not neuronal death in immature slices (Figure 4, gray column). Moreover, HSA, as well as pioglitazone and allopurinol, have no protective effect on both OGD neuronal death and on UCB-induced oxidative stress in immature hippocampal slices (Figures 3, 4), thus suggesting a different mechanism of neuronal death in these conditions.

### Effects of UCB, Pioglitazone and Allopurinol on Neuronal Death and Oxidative Stress in an *in vitro* Model of Ischemic Preconditioning ISN Immature Slices

**Figure 5:**
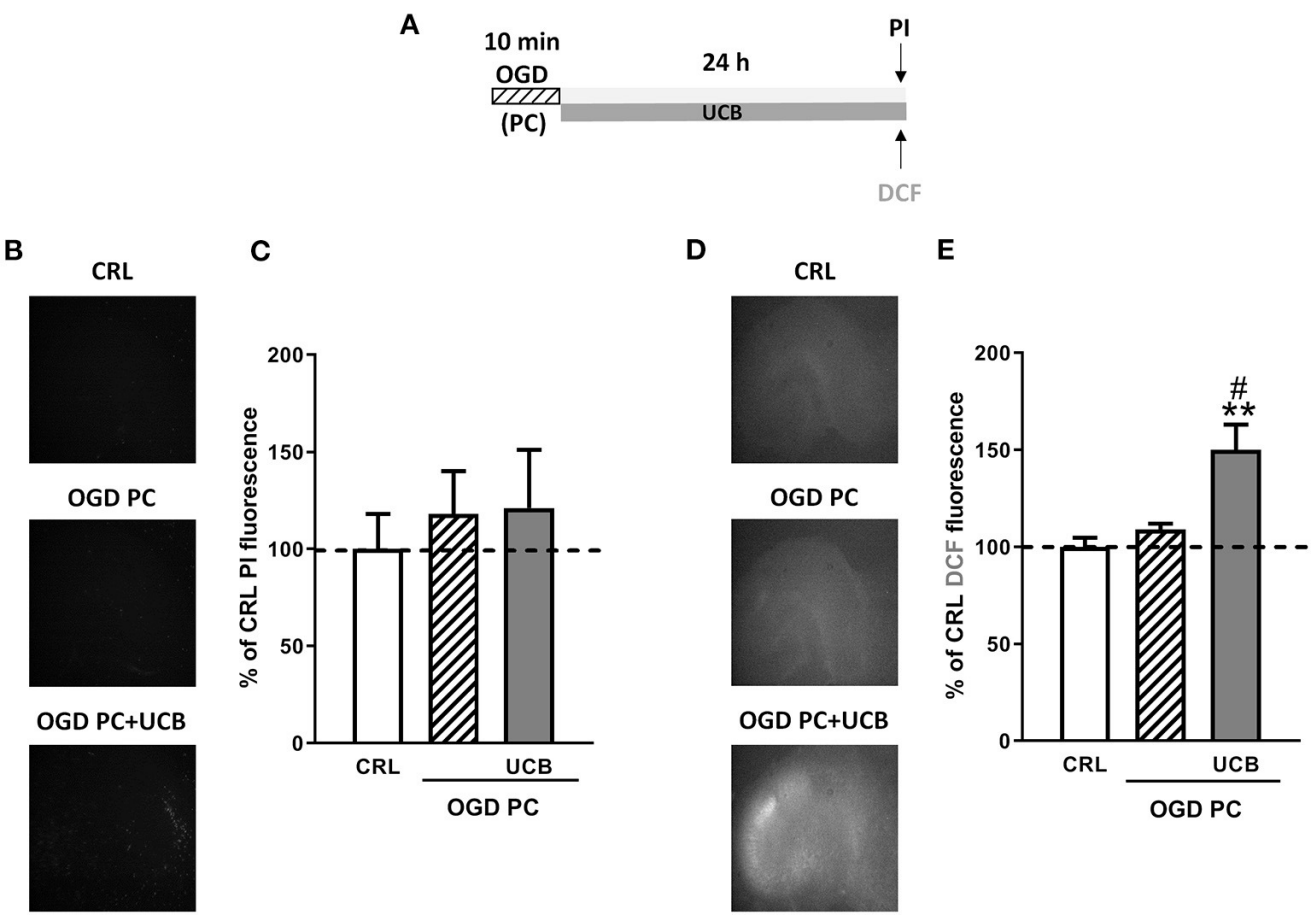
Effect of OGD-preconditioning and UCB in immature organotypic hippocampal slices. **(A)** Experimental protocol showing immature hippocampal slices subjected to 10 min OGD (preconditioning) and then to UCB (100 μM) for 24 h. **(B)** Qualitative analysis shows that OGD- preconditioning and UCB did not induce neuronal death. **(C)** Quantitative analysis is expressed as percentage of control (CRL) PI fluorescence. **(D)** Qualitative analysis shows that the addition of UCB on OGD PC induces cellular oxidative stress. **(E)** Quantitative analysis of DCFH2-DA fluorescence: Bars represent the mean ± SEM of at least seven experiments (about ≥28 slices for each experimental point). **P < 0.01 vs. CRL; ^#^*P* < 0.05 vs. OGD.

**Figure 6:**
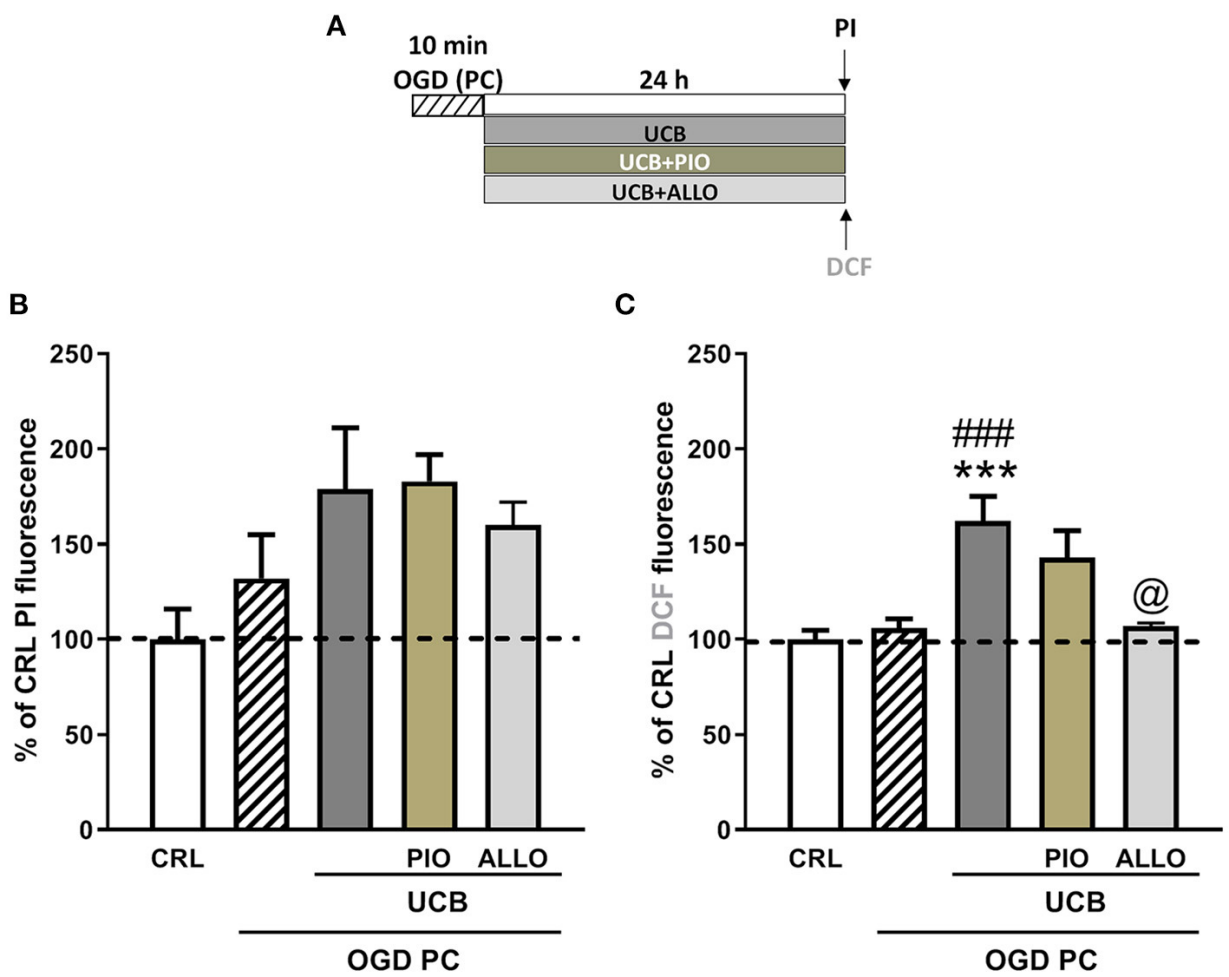
Effects of pioglitazone and allopurinol on unconjugated bilirubin (UCB) induced-toxicity in OGD preconditioned immature organotypic hippocampal slices. **(A)** Experimental protocol showing immature hippocampal slices exposed to OGD preconditioning and then to UCB (100 μM) for 24 h alone or plus pioglitazone (10 μM) or allopurinol (10 μM). Quantitative analysis shows that OGD-preconditioning, UCB, pioglitazone, and allopurinol did not affect cellular death in immature slices **(B)**, while UCB increases cellular oxidative stress that is reduced by allopurinol but not pioglitazone **(C)**. Quantitative analysis: Bars represent the mean ± SEM of at least seven experiments (about ≥28 slices for each experimental point). ***P < 0.001 vs. CRL; ^###^*P* < 0.001 vs. OGD preconditioning; ^@^*P* < 0.05 vs. UCB.

**Figure 7:**
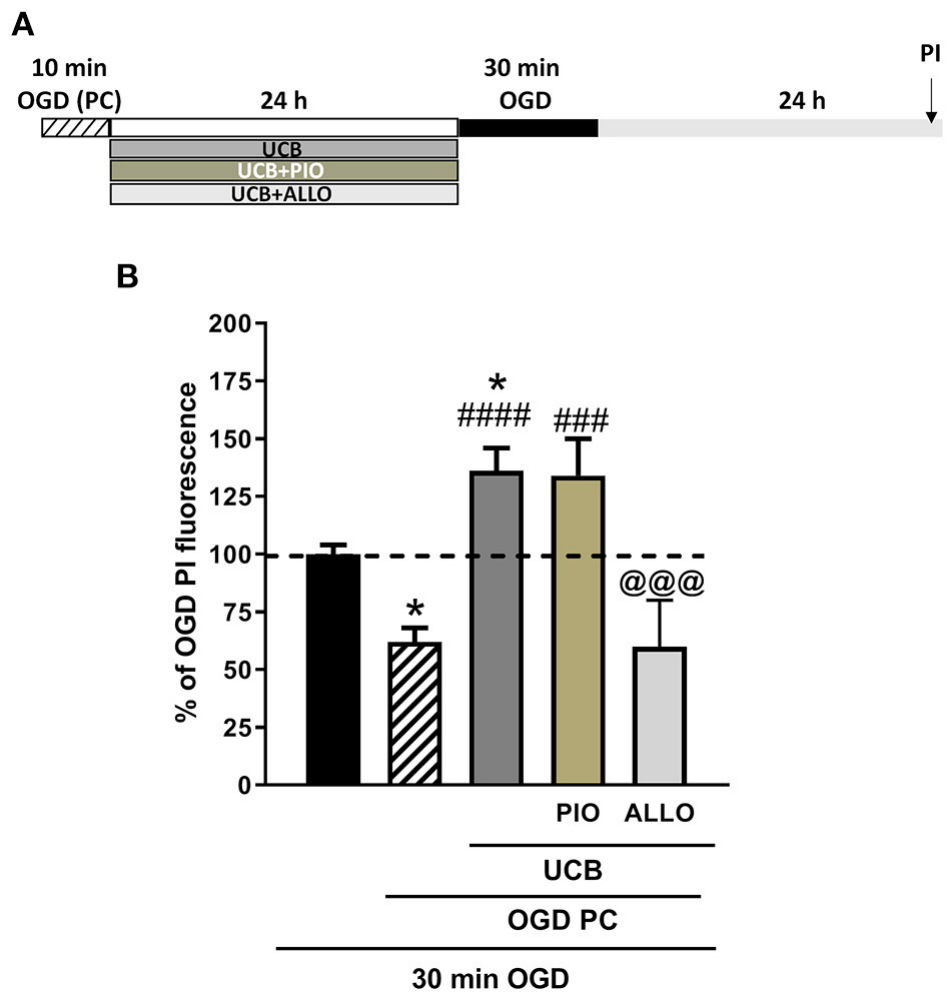
Effects of unconjugated bilirubin (UCB), pioglitazone, and allopurinol on OGD neurotoxicity in OGD preconditioned immature organotypic hippocampal slices. **(A)** Experimental protocol showing immature hippocampal slices exposed to OGD preconditioning alone or plus UCB (100 μM), pioglitazone (10 μM), and allopurinol (10 μM) for 24 h and then to 30 min OGD. **(B)** Quantitative analysis shows that OGD-preconditioning prevents cellular death in OGD exposed immature slices and that this effect is abolished by previous exposure to UCB. The addition of allopurinol but not pioglitazone is able to restore the protective effect of preconditioning. Bars represent the mean ± SEM of at least seven experiments (about ≥28 slices for each experimental point). *P < 0.05 vs. OGD; ^###^*P* < 0.001 vs. preconditioning; ^####^*P* < 0.0001 vs. preconditioning; ^@@@^*P* < 0.05 vs. UCB.

We then examined the effects of UCB, pioglitazone and allopurinol on neuronal death and oxidative stress in an *in vitro* model of ischemic preconditioning in immature slices, in order to mimic what could occur in preterm infants following IH events. Ischemic preconditioning was obtained by exposing the slices to 10 min OGD, as previously reported in Gerace et al. (35, 45, 46). For these experiments, organotypic hippocampal slices were exposed to UCB, UCB plus pioglitazone or UCB plus allopurinol immediately after 10 min OGD preconditioning and maintained for 24 h (Figure 6A).After this time, the drugs were washout and the slices were exposed to a sever OGD (30 min) and assessed for neuronal injury 24 h later (Figure 7A). As expected, we found that OGD-preconditioning did not induce cellular death and oxidative stress in immature slices (Figure 5, 6A striped column). On the contrary, the presence of UCB (100 μM) during 10 min OGD-preconditioning increased oxidative stress (Figures 5D,E) and this effect was prevented by allopurinol but not by pioglitazone (Figure 6C). Interestingly, we found that the neuroprotection against 30 min OGD induced by ischemic-preconditioning was abolished by UCB and restored by allopurinol, but not pioglitazone, probably by limiting oxidative stress (Figure 7).

## Discussion

In the present study, we investigated the effect of UCB in an “*in vitro*” model of HIE to evaluate whether it could exacerbate neuronal damage by increasing oxidative stress after HI insult. We found that UCB significantly enhances cellular death in mature slices after exposure to OGD and that this effect was abolished by equimolar amount of HSA, confirming that UCB has a role in the pathogenesis of bilirubin-induced neuronal damages (21, 43). These results are in agreement with previous studies which demonstrated that, in rat neurons, bilirubin can exacerbate the damage induced by hypoxia by reducing cell viability and facilitating glutamate-mediated apoptosis (48, 49). Moreover, we observed that UCB further increases oxidative stress induced by OGD in mature slices, suggesting that this could be one of the mechanisms which causes neuronal death. In fact, ROS have important functions in cell signaling and physiological regulations, but under pathological conditions, for istance HI and/or reperfusion–reoxygenation, they are believed to participate in tissue degenerative processes, that lead to acute and chronic brain injury (8). On the other hand, bilirubin is involved in the balance between pro-oxidant and antioxidant agents in newborn. Although many studies have demonstrated the antioxidant properties of UCB, other studies have reported that under certain conditions it can have a pro-oxidant effect (37, 38). For example, Mireles and colleagues reported that high concentration of UCB increases protein oxidation, reduced glucose-6 phosphate dehydrogenase and adenosine triphosphatase activity, and altered cell membrane integrity in red blood cells from cord blood of term infants (11). Basu et al. reported that hyperbilirubinemia can induce ROS synthesis from lipid peroxidation consistent with our results (20). On the contrary, our recent findings showed that infants with moderate-to-severe HIE present lower values of peak and mean TSB, as compared to control infants (21).

Recently, has been shown that the PPAR-γ agonist pioglitazone and the xanthine oxidase inhibitor allopurinol play an important role in attenuating neurodegenerative and neuroinflammatory events in the brain, through mechanisms involving inhibition of oxidative stress (50–52). Interestingly, in our model pioglitazone was able to decrease cellular death in OGD or UCB exposed mature slices, but was ineffective in decreasing cellular oxidative stress and in preventing UCB neurotoxicity after OGD exposure. These findings confirmed results by Xia et al. which found that pioglitazone is neuroprotective in *in vivo* and *in vitro* models of HI, decreasing pyroptosis and inducing PPARγ-mediated suppression of HGMB-1/RAGE signaling pathway, but not the reported anti-oxidant effects (25). On the other hand, allopurinol decreased both cellular death and oxidative stress in OGD or UCB exposed mature slices, confirming that its neuroprotective effect against OGD and UCB is mediated by its anti-oxidant properties (26). Indeed, allopurinol has been recently proposed as a new pharmacological intervention for neonatal brain injury and in particular for neonatal asphyxia (31–33).

Interestingly, we found that UCB did not enhances cellular death, but increased cellular oxidative stress in OGD-exposed immature slices. These results are in agreement with previous studies demonstrating that immature slices are more resistant to UCB than mature one (36–38). However, oxidative stress might cause abnormal structural changes in neurons (i.e.,: mitochondria, Golgi apparatus, etc.) which can probably prelude cellular death (37, 38). Finally, we examined the effects of UCB on ischemic preconditioning, in order to mimic what could occur after IH events mainly in preterm infants. This point is very important because IH events, which frequently occur in extremely preterm infants, might contribute to their neuroprotection against profound hypoxic events (23), especially during the 1st weeks of life when the brain is more immature (24). We found that UCB completely abolished the neuroprotection against OGD neurotoxicity induced by ischemic preconditioning. This effect is probably due to the pro-oxidant effects of bilirubin (11, 20, 37, 38), as seems to be confirmed by the fact that allopurinol, but not pioglitazone, could restore this neuroprotective effect.

In conclusion, we found that UCB increased neuronal injury and induced oxidative stress in OGD mature organotypic hippocampal slices, while induced only oxidative stress in immature ones. HSA did prevent these effects in mature slices but not in immature ones, underlining once again the different vulnerability due to the different tissue maturation, as previously shown (36–38). Pioglitazone decreased cellular death but not oxidative stress in OGD mature slices, while allopurinol decreased both of them. Pioglitazone and allopurinol had similar effects in OGD immature slices than in mature ones, but these effects were not statistically significant. Preconditioning of immature slices was protective against OGD neurotoxicity but this effect was abolished by UCB. Allopurinol was able to restore the neuroprotective effect of preconditioning against OGD, while pioglitazone was not. These results suggest that UCB can induce neurotoxicity and oxidative stress in injured brain of term and preterm infants with HIE and underline the importance of a careful management of hyperbilirubinemia in these patients.

## Data Availability Statement

The original contributions presented in the study are included in the article/Supplementary Material, further inquiries can be directed to the corresponding author/s.

## Ethics Statement

The animal study was reviewed and approved by The Italian Ministry of Health (Aut. 176; 17E9C.N.VAS) and the European Communities Council Directive of 2010/63/EU.

## Author Contributions

CD, SP, EG, and GM contributed to conception and design of the study. CD wrote the manuscript. EG performed experiments, collected data, and performed the statistical analysis. GM and EG wrote sections of the manuscript. All authors contributed to manuscript revision, read, and approved the submitted version.

## Conflict of Interest

The authors declare that the research was conducted in the absence of any commercial or financial relationships that could be construed as a potential conflict of interest.
