# Supplementary material for: Neurotoxicity of Unconjugated Bilirubin in Neonatal Hypoxic-Ischemic Brain Injury in vitro
Source: Front Pediatr. 2021 Apr 20;9:659477. doi: 10.3389/fped.2021.659477 (PMC8093500; doi:10.3389/fped.2021.659477)
Supplement: Supplementary file 1 [file Table_1.DOCX]

**Supplementary data**

**Fig. 1.** Effects of pioglitazone on OGD toxicity and unconjugated bilirubin (UCB)-induced-toxicity in mature organotypic hippocampal slices. Quantitative analysis shows that the addition of pioglitazone (10 µM) decreased cellular death in OGD and UCB exposed slices (**A and C**), but was ineffective in decreasing cellular oxidative stress (**B and D**). Quantitative analysis is expressed as percentage of control (CRL) PI (**A and C**) or DCF (**B and D**) fluorescence. Bars represent the mean±SEM of at least seven experiments (about ≥36 slices for each experimental point). *P<0.05 vs. CRL; **P <0.01 vs. CRL; ***P <0.001 vs. CRL; ^##^P<0.01 vs. OGD (ANOVA + Tukey’s w test).

**Fig. 2.** Effects of allopurinol on OGD toxicity and unconjugated bilirubin (UCB)-induced-toxicity in mature organotypic hippocampal slices. Quantitative analysis shows that the addition of allopurinol (10 µM) decreased both cellular death (**A and C**) and oxidative stress (**B and D**) in OGD and UCB exposed slices. Quantitative analysis is expressed as percentage of control (CRL) PI (**A and C**) or DCF (**B and D**) fluorescence. Bars represent the mean±SEM of at least seven experiments (about ≥28 slices for each experimental point). *P<0.05 vs. CRL; **P <0.01 vs. CRL; ***P <0.001 vs. CRL ^#^P<0.05 vs. OGD or UCB (ANOVA + Tukey’s w test).
