# Supplementary figures and images for: Neurotoxicity of Unconjugated Bilirubin in Neonatal Hypoxic-Ischemic Brain Injury in vitro
Source: Front Pediatr. 2021 Apr 20;9:659477. doi: 10.3389/fped.2021.659477 (PMC8093500; doi:10.3389/fped.2021.659477)

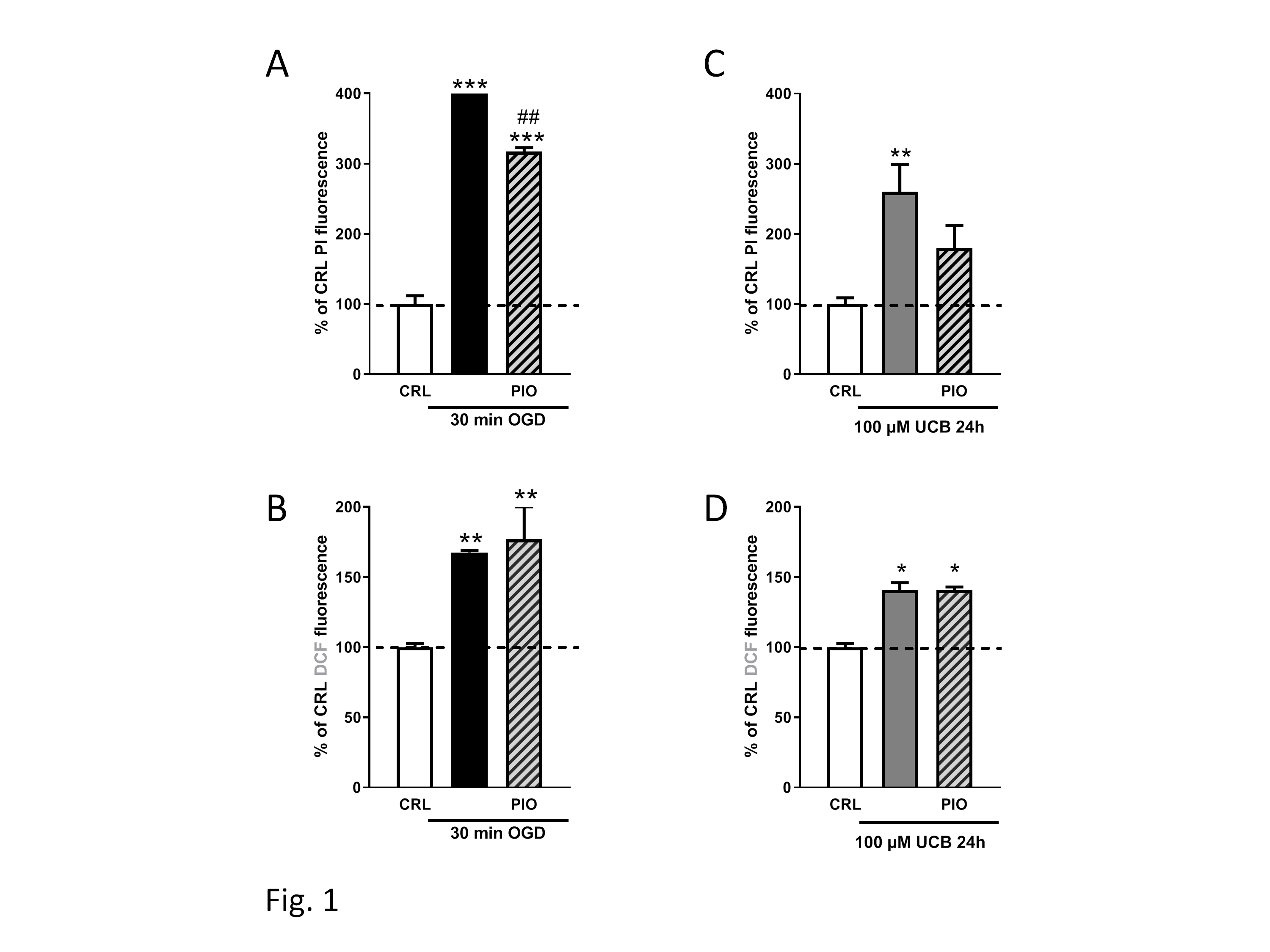

Supplement: Supplementary file 2 [file Image_1.TIF]

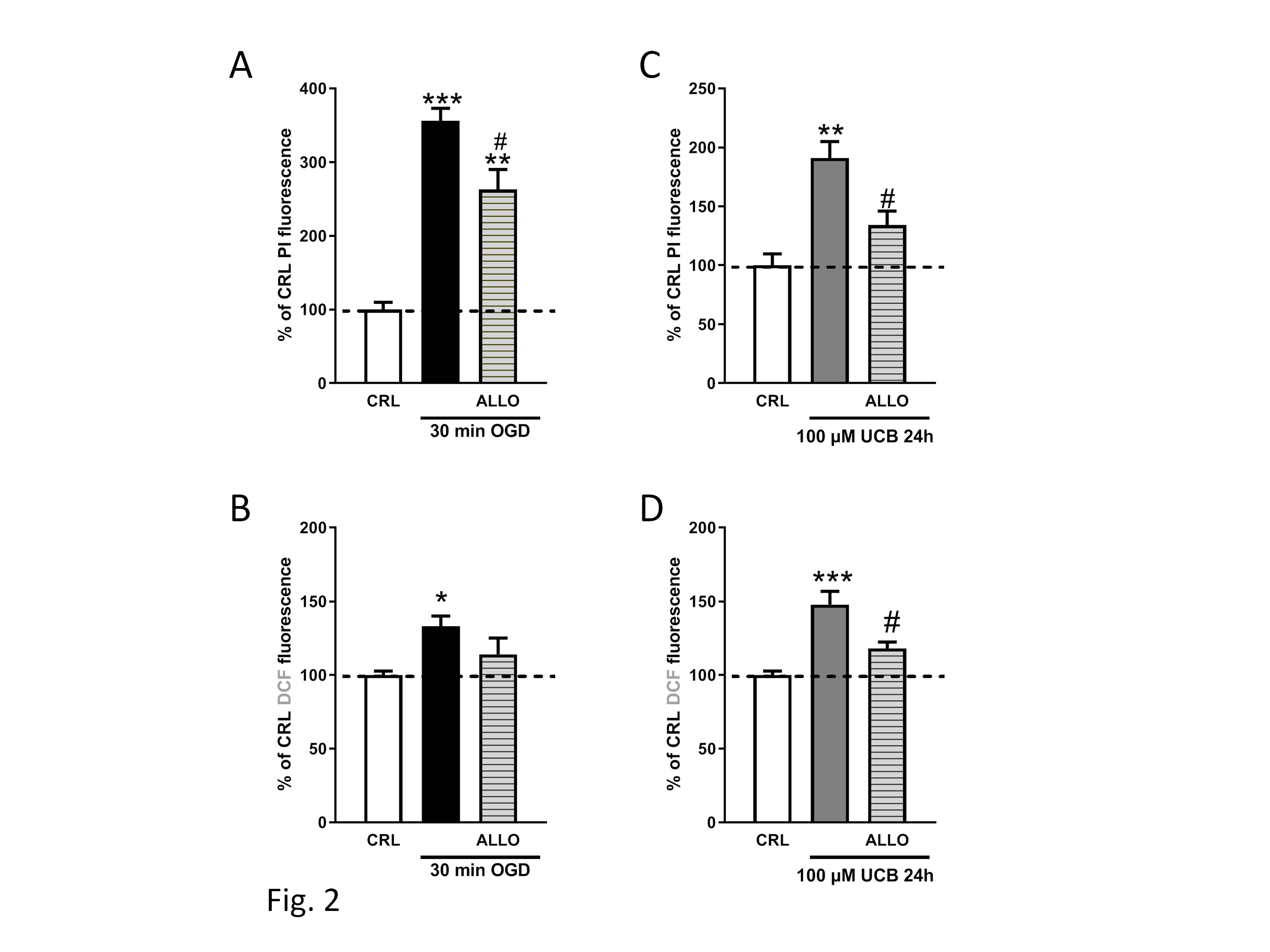

Supplement: Supplementary file 3 [file Image_2.TIF]
